# Assessment of difficulty in laparoscopic distal pancreatectomy: A modification of the Japanese difficulty scoring system – A single‐center high‐volume experience

**DOI:** 10.1002/jhbp.1010

**Published:** 2021-08-07

**Authors:** Giacomo Deiro, Matteo De Pastena, Salvatore Paiella, Alberto Balduzzi, Greta Montagnini, Elena Andreotti, Luca Casetti, Luca Landoni, Roberto Salvia, Alessandro Esposito

**Affiliations:** ^1^ Department of General and Pancreatic Surgery The Pancreas Institute University of Verona Hospital Trust Verona Italy

**Keywords:** laparoscopic pancreatectomy, learning curve, Minimally invasive distal pancreatectomy, scoring system, surgical training

## Abstract

**Background:**

The Japanese difficulty scoring system (DSS) was developed to assess the difficulty of laparoscopic distal pancreatectomy (LDP). The study aimed to validate a modified DSS (mDSS) in a European high‐volume center.

**Methods:**

Patients' clinical data underwent LDP for benign and malignant pancreatic lesion between September 2013 and February 2020 were reviewed. Expert laparoscopic surgeons performed the procedures. The mDSS consisted of seven variables, such as type of operation, malignancy, neoadjuvant therapy, pancreatic resection line, tumor close to major vessels, tumor extension to peripancreatic tissue, and left‐sided portal hypertension and/or splenomegaly. According to the difficulty level and previous score, the mDSS was subdivided into three classes: low, intermediate, and high. Surrogates of case complexity (operative time, intraoperative blood loss and blood transfusion requirements, conversion rate) were used to validate the new scoring system.

**Results:**

The study population included 140 LDP. Ninety‐five (68%), 35 (25%) and 10 (7%) patients belonged to low, intermediate, and high difficulty groups. The mDSS identified the complexity of the surgical case of the series for all the surrogates of complexity considered, namely conversion rate (*P* = .004), operative time (*P* = .033) and intraoperative blood loss (*P* = .009). No differences were recorded in the postoperative outcomes (*P* > .05).

**Conclusion:**

The mDSS for LDP better stratified the pancreatic procedures according to their complexity. The new scoring system may allow an appropriate preoperative evaluation of surgical difficulty, facilitating LDP's training program. Future prospective studies are needed to validate the mDSS.

## Graphical abstract

**Highlight**

Deiro and colleagues devised and evaluated a new difficulty scoring system to preoperatively assess the complexity of laparoscopic distal pancreatectomy based on the existing Japanese system. The modified difficulty scoring system provides appropriate preoperative assessment of surgical difficulty and facilitates the administration of training programs for laparoscopic distal pancreatectomy.

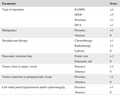

## 1 INTRODUCTION

Nowadays, the laparoscopic approach to the distal pancreatectomy (LDP) has been accepted as the gold standard treatment for resectable lesions of the pancreatic body and tail.^1^ According to the Miami international evidence‐based guidelines on minimally‐invasive pancreatic resections, minimally‐invasive distal pancreatectomy (MIDP) secured a position over open distal pancreatectomy due to better surgical outcomes (shorter hospital stay, reduced blood loss, and equivalent complication rates).^2^ Both laparoscopic and robotic approaches are safe and feasible options.^3^ Nevertheless, when ductal adenocarcinoma (PDAC) is the indication to MIDP, the procedure is a feasible, safe, and oncologically equivalent approach only in experienced hands.^4^, ^5^

The experience, surgical skills, and completion of the learning curve are still a major drawback of the widespread use of MIDP.^6^ Despite the reported training programs, there is currently no formal, universal, and standardized training program, or certified curriculum.^7^ Several studies have investigated LDP's learning curve and have used different metrics as their endpoint for proficiency.^8^, ^9^ Particularly, the primary endpoints used as surrogates for operative expertise included the operative time, conversion rate, estimated blood loss (EBL), morbidity, and hospital length of stay (LoS).^7^ Although some studies report benefits with increasing experience, not all studies have drawn a conclusion regarding the LDP’s learning curve.^10^, ^11^

A surgical procedure's complexity is influenced by different factors, such as patients' characteristics, technical issues, tumor features and location and surgeon. A difficulty scoring system (DSS) was recently developed in Japan to stratify the LDPs by surgical complexity.^12^ The DSS was previously validated, but only in Eastern Countries.^13^ Goh et al^13^ have introduced one additional point to the score for LDP performed for pancreatic malignancies in their validation of the difficulty scoring system. The revision of the DSS better stratified the complexity of the procedure, considering the diffusion of the minimally‐invasive approach for the treatment of the pancreatic cancer.

The study aims to validate a modified DSS (mDSS) in a European high‐volume center of pancreatic surgery.

## 2 MATERIALS AND METHODS

### 2.1 Study design

Patients who were submitted to LDP for benign and malignant pancreatic lesions from September 2013 to February 2020 were retrieved from the prospectively maintained institutional database and retrospectively analyzed. Clinicopathological data, intraoperative, and postoperative outcomes were collected.

Clinical parameters examined included age, gender, American Society of Anesthesiologists (ASA) score, body mass index (BMI), comorbidity, history of acute and chronic pancreatitis, neoadjuvant treatment, and any previous abdominal surgery. Tumor location, size, and vascular involvement were investigated with preoperative cross‐sectional imaging assessment. Surgical parameters, such as operative time, EBL, perioperative blood transfusion requirements, and conversion rate, were recorded. Six different surgeons performed the LDP during the study period. The learning curve was defined as achieved considering the cutoff reported in the literature, namely 17 LDP.^7^ Thirty‐ and 90‐day in‐hospital morbidity and mortality, readmission rate, and LoS were included in the analysis. The severity of complications was graded using the Clavien–Dindo classification.^14^

### 2.2 Difficulty score system

The original DSS introduced by Osaka et al included the type of operation, pancreatic resection line, the proximity of the tumor to the major vessel, tumor extension to peripancreatic tissue, and left‐sided portal hypertension/splenomegaly.^12^ Tumor location and involvement of vessels and the presence/absence of portal hypertension or splenomegaly were defined based on preoperative imaging (CT or MRI).^15^ Proximity to major vessels was defined as being within 2 cm of the splenic artery's root or splenomesenteric confluence to the pancreatic lesion. One point was added for suspected or confirmed malignancy lesion and Warshaw procedure, respectively, according to a previous validation.^13^ Recently, a large multicenter study assessed the impact of neoadjuvant therapy on the outcomes of DP.^16^ The preoperative medical treatment was associated with increased operative time, EBL, and conversion rate. Based on these results, the mDSS was created adding to Goh revised score additional difficulty points to neoadjuvant therapy before surgery (Table 1).

**TABLE 1:** Modified difficulty scoring system for distal pancreatectomy

Parameter		Score
Type of operation	RAMPS	+4
	SPDP	+3
	Warshaw	+1
	DP‐S	+1
Malignancy	Presence	+1
	Absence	0
Neoadjuvant therapy	Chemotherapy	+1
	Radiotherapy	+1
	Upfront	0
Pancreatic resection line	Portal vein	+1
	Pancreatic tail	0
Tumor close to major vessel	Presence	+2
	Absence	0
Tumor extension to peripancreatic tissue	Presence	+1
	Absence	0
Left sided portal hypertension and/or splenomegaly	Presence	+1
	Absence	0

Abbreviations: DP‐S, distal pancreato‐splenectomy; RAMPS, radical antegrade modular pancreato‐splenectomy; SPDP, spleen‐preserving distal pancreatectomy.

Each LDP was retrospectively scored from 1 to 12 by two independent HPB surgeons, where 1 and 12 were defined as the most straightforward and most challenging cases based on the DSS. Successively, the surgical procedures were stratified into three subgroups according to the DSS: low (1‐3), intermediate (4‐6), and high difficulty (7‐12).

The surrogate indicators of surgical difficulty during LDP were operative time, EBL, and conversion rate. Operative time was defined as the time interval between the skin incision and closure. EBL was defined by the amount of blood suctioned during the operation. These continuous variables were dichotomized in order to assess their correlation to the surgical complexity. A high operative time and EBL were defined as >251 minutes and >250 mL, respectively.^15^ LDP conversion to open approach was performed, after the judgement of an expert surgeon. The conversion reasons were a minimally‐invasive approach no more technically feasible or safe due to the occurrence of intraoperative unexpected events, such as the presence of a high number of intra‐abdominal adhesions or excessive intraoperative blood loss.

Postoperative outcomes, such as LoS, 90‐day hospital readmissions rate, morbidity, and mortality, were secondary indicators.

### 2.3 Surgical techniques

The institutional technique for LDP was previously described.^10^ The spleen preservation was performed selectively and only of presumed benign or uncertain biological behavior lesions.^17^ During the LDP for malignancy, the Gerota's fascia was usually removed with the specimen.^5^ The choice of the site of pancreatic transection was tailored case by case to save pancreatic tissue in benign lesions, rather than performing a standard distal pancreatectomy.^18^ The pancreatic transection was performed using two techniques only, as already reported: a triple row stapler reinforced with a PGA felt (NEOVEIL® Endo GIA™ Reinforced Reload with Tri‐Staple™ Technology 60 mm; COVIDIEN, North Haven, CT, USA) using the Purple (3 mm) or the Black (4 mm) cartridge, or an ultrasonic dissector (HARMONIC ACE®; Johnson & Johnson Medical, Ethicon) at the lowest vibration level for all duration of the pancreatic dissection.^19^ Whether to adopt one technique over the other was made at the surgeon's discretion, mostly based on pancreatic thickness. In both techniques, no additional suture was performed on the pancreatic stump or selectively on the main pancreatic duct. The handsewn management of the pancreatic stump was considered in the converted and complex cases only.

At least one surgical drain was placed close to the pancreatic remnant; when two drainages were placed, the other was put in the splenic cavity. The drain was managed in the postoperative course according to our published institutional protocol.^20^

### 2.4 Statistical analysis

The patients were divided into three groups and then compared bases on the parameters mentioned above.

Continuous variables were reported as means and standard deviation, or median and interquartile range, when pertinent. Student's *t* test and Mann–Whitney U test were used to compare continuous variables. Nonparametric tests were used when appropriate. Comparative analysis between groups was conducted using Fisher's exact tests for categorical variables. A *P*‐value <.05 was considered statistically significant (two‐tailed). Data were analyzed using Statistical Package for the Social Sciences 24.0 for Windows (SPSS, Inc.).

## 3 RESULTS

The study population included 140 consecutive patients who underwent LDP during the study period. The mDSS was applied to the study cohort, stratifying the population according to the surgical complexity. Table 2 shows the three classes were identified, such as low (n = 95, 68%), intermediate (n = 35, 25%), and high difficulty (n = 10, 7%).

**TABLE 2:** Clinicopathologic and Intraoperative data

Study Population N°= 140	Total n (%)	Low (1‐3) 95 (68%)	Intermediate (4‐6) 35 (25%)	High (7‐12) 10 (7%)	*P*‐value
Clinicopathologic data					
Age (y, DS)	55 (43‐64)	54 (44‐62)	58 (38‐64)	56 [43‐64]	.917
Sex (Female)	85 (61%)	55 (58%)	23 (66%)	7 (70%)	.565
BMI (Kg/m^2^, DS)	24 (22‐28)	25 (21‐29)	25 (23‐30)	23 (22‐28)	.260
ASA score >III	11 (8%)	9 (9%)	2 (6%)	0 (0%)	.485
Previous abdominal surgery	58 (42%)	44 (46%)	11 (31%)	3 (30%)	.259
History of Acute Pancreatitis	8 (6%)	4 (4%)	3 (9%)	1 (10%)	.540
History of Chronic Pancreatitis	6 (4%)	3 (3%)	2 (6%)	1 (10%)	.539
Preoperative lesion size	16 (9‐20)	15 (13‐20)	15 (9‐16)	20 (19‐21)	**.011**
Indication of surgery					
PDAC	20 (14%)	10 (11%)	7 (20%)	3 (30%)	.313
pNET	61 (44%)	46 (47%)	13 (41%)	2 (20%)	
IPMN	9 (6%)	7 (7%)	2 (6%)	0 (0%)	
MCN/SCN	32 (23%)	21 (22%)	6 (19%)	5 (50%)	
SPT	12 (9%)	8 (8%)	4 (12%)	0 (0%)	
Other	6 (4%)	5 (5%)	1 (3%)	0 (0%)	
Neoadjuvant therapy	5 (4%)	0 (0%)	3 (9%)	2 (20%)	.**001**
Malignancy	33 (24%)	13 (14%)	16 (46%)	4 (40%)	**<.001**
Tumor Size (mm, IQR)	29 (20‐45)	20 (24‐35)	30 (20‐40)	40 (34‐48)	.**009**
Harvest Lymph nodes (IQR)	19 (10‐26)	17 (9‐22)	20 (19‐29)	17 (14‐18)	.853
Intraoperative data					
RAMPS	8 (6%)	0 (0%)	4 (11%)	4 (40%)	**<.001**
Spleen preserving	20 (14%)	6 (6%)	14 (44%)	0 (0%)	**<.001**
Vascular resection	2 (1%)	1 (1%)	0 (0%)	1 (10%)	.**002**
Extension to peripancreatic tissue	22 (16%)	7 (7%)	8 (23%)	7 (70%)	**<.001**
Tumor close to major vessels	18 (13%)	1 (1%)	11 (31%)	6 (60%)	**<.001**
Hypertension/splenomegaly	7 (5%)	0 (0%)	0 (0%)	7 (70%)	**<.001**
Transection level					
Pancreatic neck	97 (69%)	65 (66%)	25 (78%)	7 (70%)	.**016**
GDA level	6 (4%)	2 (2%)	2 (6%)	2 (20%)	
Left aortic border	37 (27%)	31 (32%)	5 (16%)	1 (10%)	
Management Stump					
Stapler	77 (55%)	58 (59%)	14 (44%)	5 (50%)	.338
Ultrasonic scalpel	58 (41%)	36 (37%)	17 (53%)	5 (50%)	
Handsewn	5 (4%)	4 (4%)	1 (3%)	0 (0%)	
Conversion to open	26 (19%)	12 (13%)	9 (26%)	5 (50%)	.**008**
Duration of Surgery >250 min	78 (56%)	48 (51%)	22 (63%)	8 (80%)	.**033**
Associated resection	43 (31%)	34 (35%)	8 (23%)	1(10%)	.142
EBL > 250 mL	22 (16%)	10 (11%)	8 (23%)	4 (40%)	.**023**
Surgeon expertise	121 (87%)	79 (84%)	32 (91%)	10 (100%)	.242
Learning curve	52 (37%)	38 (40%)	14 (40%)	0 (0%)	.**040**

Bold indicates statistical significant value (*P* < .05).

Abbreviations: ASA: American society of Anesthesiology; BMI: body mass index; MCN: mucinous cystic neoplasm; pNET: pancreatic neuroendocrine tumor; SCN: serous cystic neoplasm; SPT: solid pseudopapillary tumor.

### 3.1 Clinicopathologic characteristics and indications for laparoscopic distal pancreatectomy

Clinicopathologic and perioperative data of the study cohort are summarized in Table 2. No significant differences were found in the baseline characteristics. The median preoperative tumor size increased proportionally to the procedure's difficulty (15 vs 15 vs 20 mm, respectively *P* =.011).

Pancreatic neuroendocrine tumor was the most frequent indication of LDP (44%), followed by cystic neoplasm (23%), and PDAC (14%). The final pathology did not differ between groups (*P* = .313).

The new variables added to the DSS was separately analyzed to assess their potential value in the prediction of the surgical difficulty. The neoadjuvant therapy was received by five patients (4%). The preoperative medical treatment was associated with increased operative time (100% vs 54%), and EBL (80% vs 13%), *P* = .035 and *P* = .002, respectively. The neoadjuvant therapy was not correlated to an increased conversion rate (20% vs 19%, *P* = .651).

Malignancy was recorded in 33 patients (24%) including 20 affected by PDAC and 13 by NET G3. The presence of malignant neoplasm was associated with higher operative time (73% vs 51%), EBL (33% vs. 10%), and conversion rate (30% vs. 15%), *P* = .021, *P* = .003, and *P* = .048, respectively.

### 3.2 Intraoperative surrogate indicators of surgical difficulty for laparoscopic distal pancreatectomy

The comparison across the study cohort groups demonstrated a correct stratification of the complexity of LDP by the mDSS (Table 2). Particularly, a higher score corresponded to an increased conversion rate (*P* = .008), longer operative time (*P* = .033) and EBL (*P* = .023). The conversion reasons differed according to the surgical difficulty. Indeed, in the low and intermediated groups, the LDPs were more frequently converted due to technical reasons, bleeding, or the presence of visceral adherences. The complex LDPs were converted often due to peripancreatic tumor infiltration, especially a posterior infiltration, with an unsafe vascular control.

During the study period, six surgeons performed the LDP: two had completed the learning curve before the study, two completed it during the study period and the remaining were still in training. They carried out 69, 52 and 19 LDPs, respectively. Excluding the cases performed by the two operators who have not yet completed the training at the end of the study, the surgeon's expertise did not affect the results (*P* = .242). As expected, the surgeons started and completed the learning curve with a progressive increase of surgical difficulty (*P* = .040).

**FIGURE 1:**
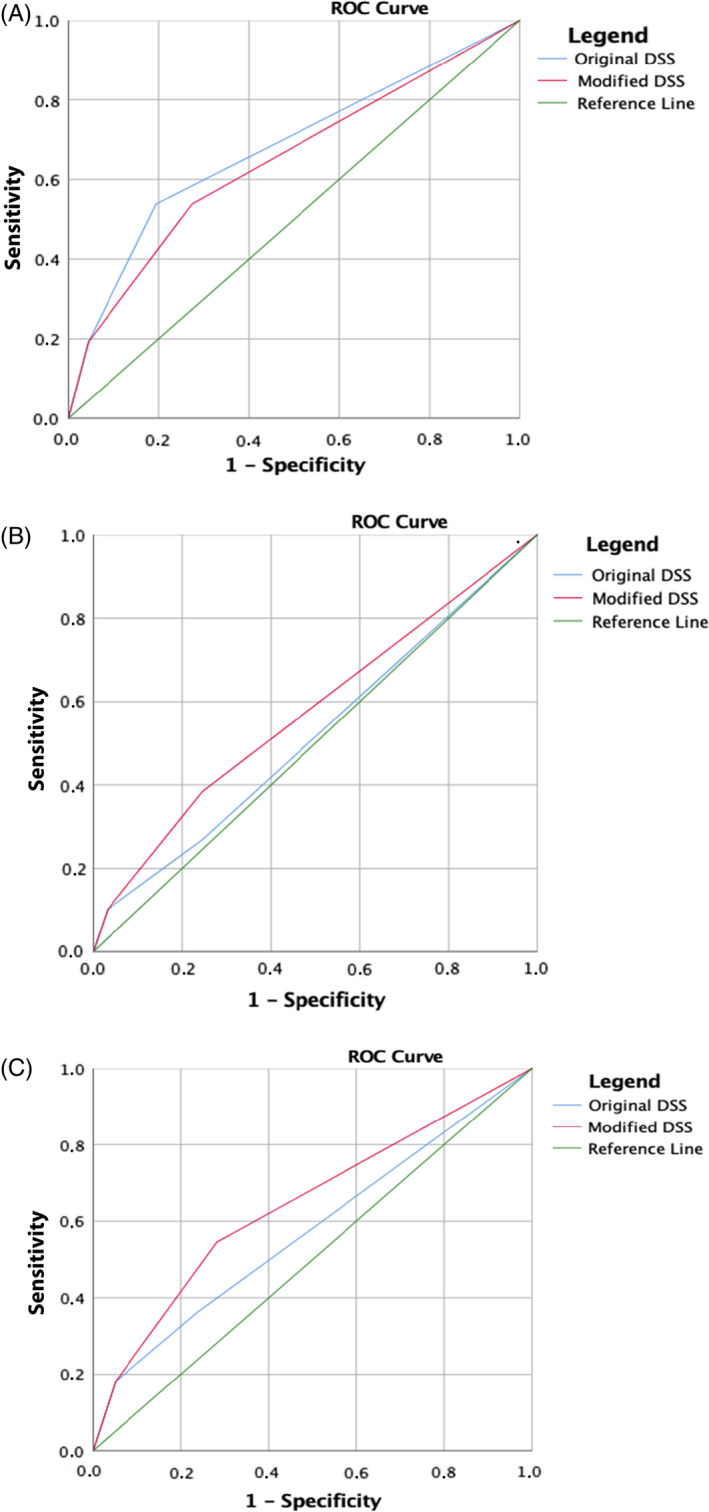
(A) Conversion rate: AUC DSS: 0.679 (95% CI: 0.55‐0.80); AUC mDSS: 0.647 (95% CI: 0.52‐0.77). (B) Operation time: AUC DSS: 0.520 (95% CI: 0.42‐0.61); AUC mDSS: 0.576 (95% CI 0.48‐0.67). (C) Estimated blood loss: AUC DSS: 0.575 (95% CI: 0.43‐0.71); AUC mDSS: 0.643 (95% CI: 0.51‐0.78)

The mDSS was compared to the original DSS using the ROC curves, as shown in Figure 1. The surrogate indicators of surgical difficulty were used to assess the reliability of the mDSS and evaluate the influence of the additional parameters. The mDSS had an acceptable prediction of the LDP difficulty, superior to the original score.

### 3.3 Postoperative data

The surgical outcomes are reported in Table 3. The overall postoperative complications rate was 59% (82 patients), of whom 9%, (13 patients) had a Clavien‐Dindo score ≥3 complications.

**TABLE 3:** Postoperative data

Study Population N = 140	Total n (%)	Low (1‐3) 95 (68%)	Intermediate (4‐6) 35 (25%)	High (7‐12) 10 (7%)	*P*‐value
Any complication	82 (59%)	55 (57%)	21 (60%)	6 (60%)	.960
Clavien‐Dindo >3	13 (9%)	6 (6%)	5 (17%)	2 (20%)	.132
POPF					
Grade B	31 (22%)	22 (23%)	6 (17%)	3 (30%)	.542
Grade C	2 (2%)	2 (2%)	0 (0%)	0 (0%)	
DGE	2 (1%)	1 (1%)	1 (1%)	0 (0%)	.633
PPH	16 (11%)	12 (12%)	2 (6%)	2 (20%)	.441
Chyle leak	2 (1%)	1 (1%)	0 (0%)	1 (10%)	.055
Abdominal collection	42 (30%)	30 (31%)	10 (31%)	2 (20%)	.772
Length of Stay (d, IQR)	8 (7‐10)	8 (7‐11)	7 (7‐10)	8 (6‐19)	.995
Reintervention	9 (6%)	5 (5%)	3 (9%)	1 (10%)	.619
Readmission	15 (11%)	7 (8%)	7 (02%)	1 (10%)	.158

Abbreviations: POPF: postoperative pancreatic fistula; PPH: Post pancreatectomy hemorrhage,

No differences were observed between the groups regarding the pancreatic specific complications (all *P* > .05). Similarly, no statistical differences were found regarding the secondary surrogate indicators. Particularly, LoS (8 vs 7 vs 9 days, *P* = .995), reintervention rate (5% vs 9% vs 10%, *P* = .619), and readmission rate (9% vs 22% vs 10%, *P* = .158) were comparable. The mortality rate of the series was 0%.

## 4 DISCUSSION

The new mDSS showed to be a useful tool, able to correctly stratify the LDPs according to their complexity. If further confirmed, this scoring system may help to implement the stepwise approach to LDP with a safe surgical training, better preoperative assessment and counseling of the patients.

The MIDP is a well‐standardized procedure, but it can also be associated with a wide range of operative challenges.^4^, ^21^, ^22^ No objective criteria and no definitions of grades of complexity are currently available, weakening the MIDP’s surgical training. The lack of reliable tools to define the degree of the complexity of LDP is responsible for the diffusion of non‐standardized training programs in MIDP.^8^, ^23^, ^24^

Although LDP's learning curve is by definition a dynamic process, the concept of its completion, reaching a cutoff of procedures, is somehow static and misleading. Indeed, the cutoffs proposed, ranging from 10 to 40 LDP,^7^, ^15^, ^25^ are supposedly based on a standard and not always a simple procedure. Surely none of the studies has mentioned the grade of difficulty of the procedures. Reasonably, most MIDPs of the learning curve were influenced by the complexity of the cases.

The most commonly used metric to assess the operative proficiency of LDPs is the reduction of operative time.^15^ The duration of the surgery can be influenced by different factors, and can especially be associated with the surgical skill of the operator. However, the present study also confirmed the correlation between the operative time and complexity of the pancreatic resection. The study results demonstrated that the judgment of the surgical skill and the level of the learning curve of the pancreatic surgeon cannot be left out of consideration in the conversion rate and EBL. The use of these indicators as surrogates of surgical difficulty of these parameters has been widely reported in the literature.^26^, ^27^ The preoperative patient's characteristics, particularly the body mass index, did not result as additional factors of the complexity of the surgical procedure.^28^ Notably, the postoperative outcomes were not influenced by the surgical complexity based on the mDSS, in contrast with previous studies.^9^, ^29^ This result can be explained by the surgical skills of the surgeons that performed this series. Indeed, the study cohort did include a surgical training program to evaluate the evolution of the ability of the surgeons.

The DSS of LDP developed by Ohtsuka et al was created in 2017 using a survey involving four expert surgeons and it was based on 80 LDPs.^12^ This score did not consider the diffusion and increment of the use of MIDP in the treatment of pancreatic malignancies. The widespread use of neoadjuvant therapy (even for resectable lesions) had increased the center that have approach PDAC minimally‐invasive. Recently, a large multicenter study assessed the impact of neoadjuvant therapy on the outcomes of DP.^16^ Preoperative medical treatment was associated with increased operative time, EBL, and conversion rate. The study results confirmed the association of the neoadjuvant therapy and the presence of a malignant neoplasm with an increase in the surrogate indicator of surgical difficulty. Therefore, these parameters must be contemplated during evaluation of the surgical complexity of the MIDP due to the potential addition of complications, even for expert surgeons.^16^ Patient selection seems to be crucial in a safe stepwise approach to the MIDP. The reach of a complete learning curve and surgical laparoscopic skills allows an increase in the difficulty of MIDP while minimizing the danger.^30^

Some potentially strengths and applications of the mDSS can be considered. First, the patient's stratification based on the surgical difficulty can ultimately improve the patient selection, according to the center's surgeon staff's surgical skills. The tricky cases can be identified and shared with referral centers or performed with an appropriate proctoring. Second, the scoring system may allow the standardization of the surgical training program for LDP. A stepwise approach to the LDP can be created, generating a curriculum or license based on the surgical skills reached. Third, the mDSS could be used to compare data from the literature, objectifying the surgical techniques' results, and for difficulty‐adjusted comparisons. Fourth, the subsequent studies dealing with LDP’s learning curve may include the mDSS to go deep into the training details. Fifth, the mDSS could improve the preoperative assessment and counseling of the patient.

Some limitations have to be considered as well. First, the retrospective analysis of the series can generate a bias. Second, even if all the participating surgeons have high expertise in LDP, different levels of surgical skills could have impacted the perioperative outcomes. Third, the introduction of an additional point for the neoadjuvant treatment was applied considering the judgment of expert minimally‐invasive surgeons and should be validated in a large cohort.

## 5 CONCLUSION

The mDSS for LDP better stratified the pancreatic procedures according to their complexity. The new scoring system may allow an appropriate preoperative evaluation of surgical difficulty, facilitating LDP's training program. Future prospective studies are needed to validate the mDSS.

## CONFLICT OF INTERESTS

The authors have no conflict of interest.
